# Autosomal dominant tubulointerstitial kidney disease genotype and phenotype correlation in a Chinese cohort

**DOI:** 10.1038/s41598-020-79331-w

**Published:** 2021-02-11

**Authors:** Kunjing Gong, Min Xia, Yaqin Wang, Na Wang, Ying Liu, Victor Wei Zhang, Hong Cheng, Yuqing Chen

**Affiliations:** 1 Renal Division, Department of Medicine, Peking University First Hospital, Beijing, 100034 China; 2 Institute of Nephrology, Peking University, Beijing, 100034 China; 3 Key Laboratory of Renal Disease, Ministry of Health of China, Beijing, 100034 China; 4 Key Laboratory of Chronic Kidney Disease Prevention and Treatment, Ministry of Education, Beijing, 100034 China; 5 AmCare Genomics Laboratory, Guangzhou, China; 6 Baylor College of Medicine Department of Human and Molecular Genetics, Houston, USA; 7 Division of Nephrology, Beijing AnZhen Hospital, Capital Medical University, Beijing, 100029 China

**Keywords:** Disease genetics, Interstitial disease

## Abstract

Genes of *UMOD*, *HNF1B*, *MUC1*, *REN* and *SEC61A1* were reported to be associated with autosomal dominant tubulointerstitial kidney disease (ADTKD). 48 probands and their family members (N = 27) were enrolled in this genetic screening study. A combination of methods was employed for comprehensive molecular analysis of both copy number variations (CNVs) and single nucleotide variants (SNVs). 35 probands were followed for years. The phenotype-genotype and genotype-outcome correlation were inferred from these datasets. In this cohort, 18 probands were diagnosed with ADTKD, according to Kidney Disease: Improving Global Outcomes (KDIGO) guideline. Moreover, 11 probands were diagnosed with ADTKD-UMOD, one with ADTKD-REN and one with ADTKD-HNF1B, based on molecularly confirmed pathogenic variants. The 11 *UMOD* variants were mainly located in codons 28 to 289 and half of the variants were found to change the cysteine amino acid. According to the follow-up data, suspected ADTKD individuals had a better prognosis compared to ADTKD individuals (p = 0.029). Individuals with a cysteine substitution in the *UMOD* gene appeared to have a better prognosis than individuals with other amino acid substitutions (p = 0.015).

## Introduction

Autosomal dominant tubulointerstitial kidney disease (ADTKD) is considered to be one of the common causes of end stage renal disease (ESRD), especially in people whereby familial aggregation is observed^1^. ADTKD has been reported using different names, including familial juvenile hyperuricemic nephropathy (FJHN), medullary cystic kidney disease (MCKD) and maturity-onset diabetes mellitus of the young type 5 (MODY5)^2–4^. In terms of genetic etiologies, several genes, including *UMOD* (OMIM 16p12.3 and 191845)^2^, *REN* (OMIM 1q32.1 and 179820)^5^, *HNF1B* (OMIM 17q12 and 189907)^4^ and *MUC1* (OMIM 1q22 and 158340)^3^ have been confirmed to be responsible for some forms of ADTKD. A recent study reported that *SEC61A1* (OMIM: 609213) might be considered as a new disease-causing gene of ADTKD^6^. Variants in *UMOD, REN, HNF1B, and SEC61A1* genes can be detected by DNA sequencing. In addition, gross deletions or duplications in *HNF1B* need to be detected by multiplex ligation-dependent probe amplification (MLPA), or quantitative PCR. Genotyping of *MUC1* remains challenging because of the complex gene structure. A *MUC1* mutation was described as a cytosine insertion in the extracellular variable number tandem repeat (VNTR) domain of the gene which led to a frameshift change^3,7^. Other insertion and deletion mutations (indel) in the VNTR region of *MUC1* have also been described as pathogenic variants^8^.

The prevalence of ADTKD and subtypes remain unknown. Most of the ADTKD individuals have been clinically diagnosed as having chronic kidney disease (CKD) without a clear molecular diagnosis due to latent symptoms, mild urinary sediment, and atypical pathological lesions. In this study, we screened for variants in five genes associated with ADTKD by a combination of methods, analyzed the phenotype-genotype correlation and genotype-outcome correlation in a Chinese cohort.

## Results

### General clinical features of the cohort

In total, 18 patients were diagnosed with ADTKD according to the criteria of the KDIGO guideline^1^. Among them, clinically significant genetic variants were identified in 13 probands, and 5 probands were clinically diagnosed ADTKD without identifying pathogenic variants in known genes associated with ADTKD (Table 1). In addition to the clinically diagnosed ADTKD patients, there were 30 probands with suspected ADTKD, because of either positive family history or pathological data. When the biochemical traits, family history, and demographic information were examined, no statistical difference was found between the confirmed ADTKD and suspected ADTKD groups in terms of average age, male/female ratio, serum creatinine level, serum uric acid level, renal cysts identification and histological data (Table 1).

**Table 1:** Clinical features of the cohort.

Characters	The whole cohort	ADTKD	Suspected ADTKD	P value
Number	48	18	30	
Male/Female	25/28	9/9	14/16	0.823
Age of diagnosis	30.9 ± 12 (13,68)	30 ± 10.1	31.4 ± 13.6	0.689
Creatinine (μmol/L)	195 ± 114 (45,717)	203.3 ± 81	190.1 ± 130.9	0.786
Uric acid (μmol/L)	487.8 ± 142.2 (187,781)	521.1 ± 150	467.9 ± 136	0.213
Blood Urea Nitrogen (mmol/L)	7.8 ± 5.2	8.2 ± 5.6	7.5 ± 4.9	0.674
24-h urinary protein (g)	0.38 ± 0.55	0.27 ± 0.36	0.46 ± 0.63	0.265
With/without Proteinuria (No.)	8/40	3/15	5/25	1.000
Bland urine sediment (No.)	48	18	30	1.000
Gout	13	5	7	1.000
Hypertension	18	6	12	0.808
**Renal cyst**	19	7	12	0.939
Unilateral	3	2	1	
Bilateral	16	5	11	
**Kidney size**				
Normal	39	16	23	0.504
Small(< 9 cm)	9	2	7	
Family history	62.5%	83.3%	50%	***0.045***
**Renal biopsy**	15	9	6	0.064
Tubulointerstitial-nephropathy	12	7	4	
Medullary cystic kidney disease	2	2	1	
Cystic dilation of tubules/acute tubular injury	1	0	1	
Number of patients with genetic variation	13	13	0	

*Family history* Family history positive means there were other family members with renal disease or hyperuricemia, *ADTKD* autosomal dominant tubulointerstitial kidney disease.

### Mutational profiles of molecular genetic analysis

We utilized a combination of Sanger, next-generation sequencing (NGS), and MLPA (Supplementary figure S1) to detect SNVs and CNVs in 5 genes associated ADTKD and whole exome sequencing (WES) to identify new candidate genes. Briefly, we sequenced the four genes (*UMOD/REN/HNF1B/SEC6lA1*) by Sanger sequencing, CNVs of *HNF1B* by MLPA, and sequenced *MUC1* by long-range high-fidelity PCR combined with NGS. We performed WES in patients without pathogenic variants in the 5 genes associated with ADTKD.

No pathogenic variants were detected in *MUC1* and *SEC6lA1* in any of the patients. 12 missense changes and 1 frameshift mutation were detected in 13 probands. Among them, only 10 individuals had a family history of CKD or hyperuricemia. 11 probands were confirmed to have significant variants in *UMOD*. Moreover, 1 proband had compound heterozygous variants in *REN* and another was heterozygous for a change in *HNF1B*. For variants in *UMOD*, five of the missense variants were predicted to change a cysteine residue (p.Cys35Tyr, p.Cys112Gly, p.Cys248Trp, p.Cys287Phe, and p.Cys347Arg), and six variants resulted in the replacement of other residues (p.Asn38Ile, p.Leu66Pro, p.Val109Glu, p.Cys112Gly, p.Pro236Gln, and p.Arg385Trp) (Supplementary figure S2). The p.Asn38Ile change may affect a potential N-glycosylation site^9^. In addition, the *REN* change in family 12 was p.Lys32Thr which was located in exon 1 as previously reported^10,11^ and the *HNF1B* change in family 13 was a p.Arg295His in exon 4 (Table 2).

**Table 2:** Detected variants and corresponding clinical outcome.

No	Family history	Gene	Nucleotide change	Effect on peptide sequence	ACMG	Gender	Diagnosis age	Creatinine μmol/L	Uric acid μmol/L	Renal cyst	Interstitial `fibrosis	Treatment	Follow-up (year)	ESRD (yes/no)/age
F1	No	*UMOD*	c.104G > A	p.Cys35Tyr	Pathogenic	Male	20	163	749	No	NA	XO inhibitors	4	No/24
F2	No	*UMOD*	c.1039 T > C	p.Cys347Arg	Pathogenic	Female	22	154	691	No	Yes	XO inhibitors	5	No/27
F3	Yes	*UMOD*	c.113A > T	p.Asn38Ile	Pathogenic	Female	38	155	253	No	NA	No	2	Yes/40
F4	Yes	*UMOD*	c.197 T/C	p.Leu66Pro	Pathogenic	Male	44	203	380	Yes	Yes	BP-lowering drugs	5	No/49
F5	Yes	*UMOD*	c.272delC	p.Ser91	Pathogenic	Male	50	317	414	Yes	NA	BP-lowering drugs	2	Yes/52
F6	No	*UMOD*	c.326 T/A	p.Val109Glu	Pathogenic	Male	18	195	489	No	NA	XO inhibitors	2	Yes/20
F7	Yes	*UMOD*	c.334 T > G	p.Cys112Gly	Pathogenic	Female	41	250	540	No	NA	XO inhibitors	0	No/41
F8	Yes	*UMOD*	c.707 C/A	p.Pro236Gln	Pathogenic	Female	24	230	606	No	Yes	XO inhibitors + BP-lowering	2	Yes/26
F9	Yes	*UMOD*	c.744C/G	p.Cys248Trp	Pathogenic	Female	21	136	433	Yes	NA	No	5	No/26
F10	Yes	*UMOD*	c.860G. > T	p.Cys287Phe	Pathogenic	Female	41	182	474	Yes	NA	BP-lowering drugs	4	No/45
F11	Yes	*UMOD*	c.1153C > T	p.Arg385Trp	Likely pathogenic	Male	22	469	781	No	Yes	XO inhibitors	3	Yes/25
F12	Yes	*REN*	c.95A > C	p.Lys32Thr	Uncertain significance	Male	34	158	617	Yes	NA	XO inhibitors + BP-lowering	1	No/35
F13	Yes	*HNF1B*	c.884G > A	p.Arg295His	Likely pathogenic	Male	34	158	484	No	NA	XO inhibitors + antidiabetic drugs	4	No/38

*F* family, *NA* not available, *XO inhibitors* xanthine oxidase inhibitor, allopurinol or febuxostat, *BP-lowering drugs* blood pressure lowering drugs, *ESRD* end stage renal disease.

Pathogenicity of the identified variations was evaluated according to the American college of medical genetics and genomics (ACMG) standards and guidelines. For analysis, we examined the frequency of the variants in 1000 genome browser and human gene mutation database. In addition, we sequenced 92 healthy donors to obtain variants found in healthy individuals. Function study of variants in *UMOD* has been performed in our previous study except for p.Cys112Gly. Also, we evaluated the pathogenicity of missense variants with Polyphen and other software. One frameshift variant and nine missense variants can be classified as “pathogenic”. One missense variant in *UMOD* (p.Arg385Trp) and one missense variant in *HNF1B *(p.Arg295His) can be classified as “likely pathogenic”, while the missense variant of *REN* (p.Lys32Thr) can only be classified as “uncertain significance” (Table 2). One missense variant in *HNF1B* (p. Arg295His) identified in our cohort also report in another study^12^. We did not detect CNVs in *HNF1B* (Supplementary figure S3) and *MUC1* variants in our cohort. Details of the ACMG evidence were presented at supplementary table S2.

WES was performed on 18 probands (4 clinically identified ADTKD and 14 suspected ADTKD). All of them had a positive family history of CKD, or hyperuricemia or both. We did not identify any potential candidate genetic variants associated with clinical features (Supplement table S3).

### Genotype and phenotype correlation in the cohort with ADTKD

In the cohort, 35 patients including 13 genetically confirmed ADTKD, 2 ADTKD-NOS (ADTKD-not otherwise specified; clinically confirmed ADTKD), and 20 ADTKD suspected were followed during the year 2012 to 2018. Thirteen ADTKD and 11 individuals with suspected ADTKD received the proper medical management based on the appropriate treatment guidelines^13^. Two individuals with ADTKD and 9 individuals with suspected ADTKD presented with only minor symptoms and are being followed annually without treatment.

**Figure 1:**
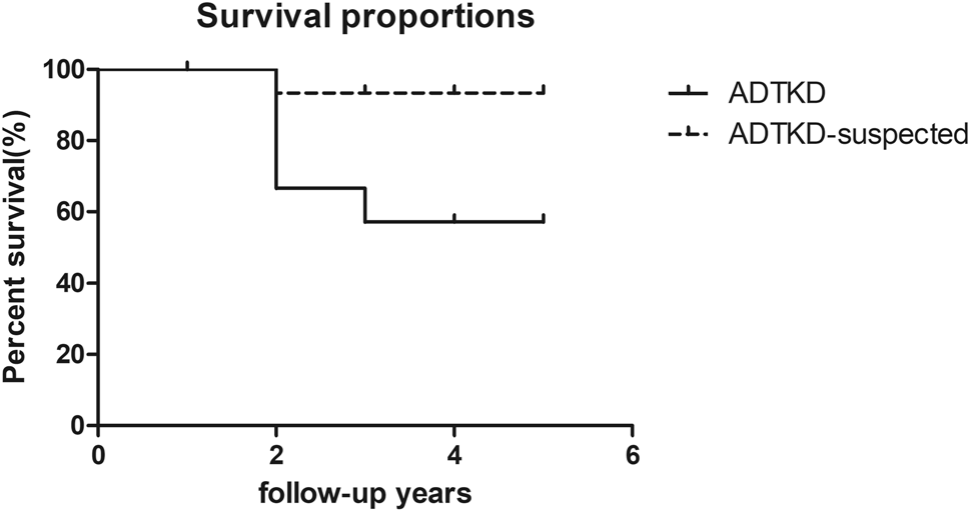
Kaplan–Meier survival analysis of ADTKD and ADTKD-suspected. Renal survival of ADTKD and ADTKD-suspected was investigated by Kaplan–Meier analysis. The longest follow-up time was in the fifth year. Five of 15 ADTKD patients developed ESRD compared to one of 20 ADTKD-suspected (p = 0.029) patients.

Among the 13 genetically confirmed ADTKD, the person with a *REN* mutation recovered in one year and the proband with a *HNF1B* variant remained stable. 5 out of the 10 ADTKD-UMOD progressed to ESRD (Table 2), whereas only one person progressed to ESRD among ADTKD suspected. Kaplan–Meier survival analysis indicated that individuals with suspected ADTKD had a better prognosis compared to the ADTKD individuals (p = 0.029, Fig. 1). Detailed clinical descriptions of the follow-up participants were shown in supplementary table S4.

The 11 *UMOD* variants located in exon 4 and exon 6. Six of the eleven (55%) *UMOD* variants were located in the three epidermal growth factor (EGF)-like domains (p.Cys35Tyr and p.Asn38Ile in the first, p.Leu66Pro and p.Ser91 in the second, and p.Val109Glu and p.Cys112Gly in the third EGF-like domain). Three of the eleven (27.2%) variants, p.Pro236Gln, p.Cys248Trp and p.Cys287Phe, located in the D8C region. Two variants (p.Cys347Arg, p.Arg385Trp) located in the zona pellucida (ZP)-N domain (Supplementary Figure S2). EGF-like domains might play important roles in protein–protein interactions while ZP domain might be essential for protein polymerization^14^. We grouped the variants into three classes based on domains and compared the outcomes of the individuals with these variants, but no differences were observed (Table 3A). Additionally, we divided the individuals with UMOD variants into two groups based on whether the variation was a cysteine substitution because more than half were cysteine changes. Individuals with cysteine substitution appeared to have a better prognosis compared to individuals with another amino acid substitution (Table 3B).

**Table 3:** Renal survival of ADTKD-UMOD patients classified by UMOD domain or cysteine substitution.

(A) Domain	EGF	D8C	ZP	Total
N	6	3	2	11
Male/female	4/2	0/3	1/1	5/6
Prognosis ESRD (age)	3 (40,52,20)	1 (26)	1 (25)	5
Slowly progression(age)	3 (24,41,44)	2 (26,45)	1 (27)	5

B	Cysteine	Others	Total	
N	5	6	11	
Male/female	1/4	4/2	5/6	
Diagnosis age	29 ± 11	33 ± 13	31 ± 12	
Prognosis ESRD (age)	0	5 (20,25,26,40,52)	5	
Slowly progression (age)	5 (24,26,27,41,45)	1 (49)	6	

*ADTKD* autosomal dominant tubulointerstitial kidney disease, *NOS* not otherwise specified, *ESRD* end stage renal disease, *EGF* epidermal growth factor, *ZP* zona pellucida.

A. Renal survival of ADTKD-UMOD patients classified by *UMOD* domain. The prognosis was analyzed by Kaplan–Meier analysis, with p > 0.05. B. Renal survival for ADTKD-UMOD patients classified by cysteine substitution. Diagnosis age did not statistically differ between the two groups. (p = 0.632) The prognosis was analyzed by Kaplan–Meier analysis, with P = 0.015. *ESRD* end stage renal disease.

**Figure 2:**
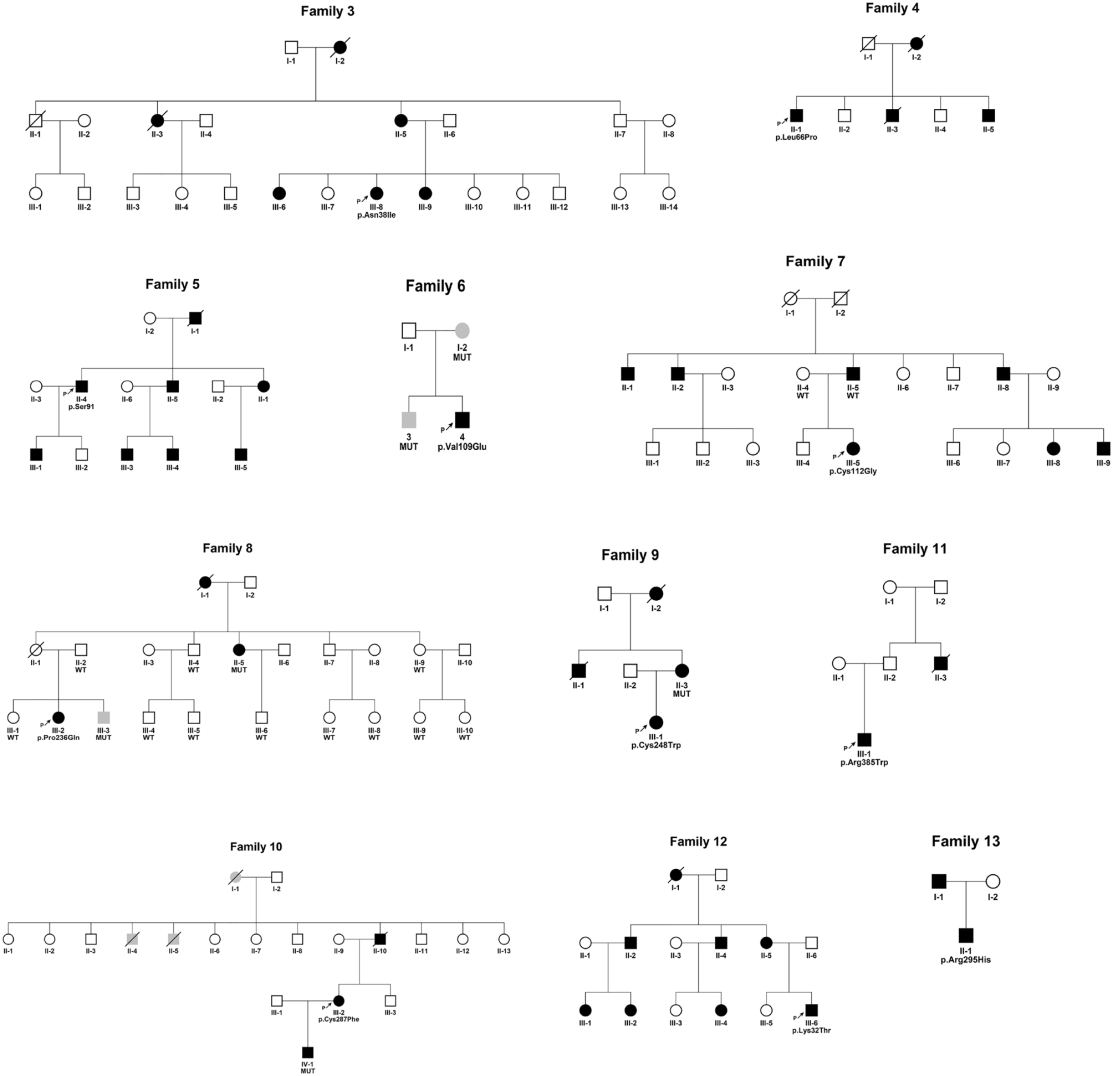
Pedigree information of the families. Proband from family 3–13 had a positive family history of ADTKD. Black symbols denoted clinically affected individuals. Open symbols denoted clinically unaffected individuals. Gray symbols denoted family members with variants but no clinical symptoms. Arrow denoted the proband and below the proband symbol are the amino acid symbols. Family 3, family 12 and family 13 had variants in genes *UMOD, REN,* and *HNF1B,* respectively. For the family members which genetic testing was performed the results were listed below the symbol. *MUT* Family members carried the same clinically significant variant as the proband, *WT* Individuals without pathogenic variants.

We also analyzed the association between clinical data and genetic variations. All probands with *UMOD/REN/HNF1B* variants had a clinical history of hyperuricemia and increased plasma creatinine, and the proband with *HNF1B* variation also presented with diabetes mellitus, hypokalemia and an abnormal liver test (Table 2). The patient with a *REN* mutation had a positive family history of renal cysts. Most of ADTKD-UMOD individuals presented with family histories of hyperuricemia or CKD. In family 6, the mother and the elder brother of the proband carried the same heterozygous variant of *UMOD,* without hyperuricemia or CKD (Fig. 2). Incomplete penetrance may explain this phenomenon as previously reported^15,16^. We also identified UMOD variants in family 1 and family 2 who had negative family history. Proband of family 1 had an increased plasma uric acid (749 μmol/L) and creatinine (163 μmol/L) at the time of diagnosis and his plasma creatinine increased to 242 μmol/L after 4 years. His parents had kept normal plasma creatinine and uric acid levels during the 4 years. Proband of family 2 had an increased uric acid 691 μmol/L and creatine 154 μmol/L at diagnosis and progressed for 5 years, while her parents presented no signs of CKD or hyperuricemia. We did not get permission to do genetic tests for the parents of family 1 and family 2. We cannot confirm whether the UMOD variants in family one and 2 are de novo or incomplete penetrance. Thus it is difficult to give an exact rate of incomplete penetrance and compared to other studies^15,16^.

**Figure 3:**
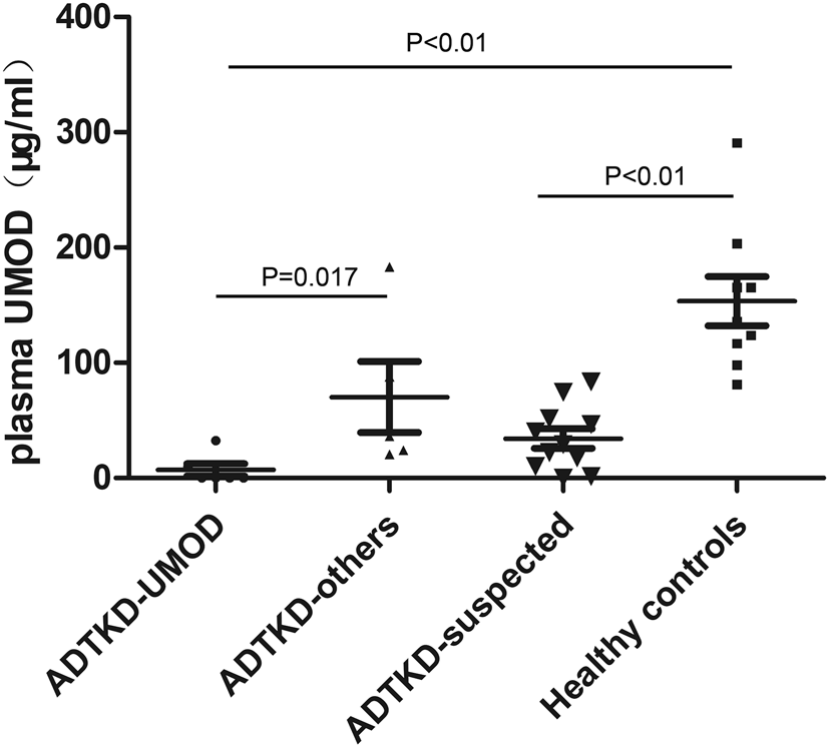
Uromodulin concentration of the individuals with suspected ADTKD in plasma. ADTKD-UMOD: ADTKD individuals with UMOD variants, n = 6; ADTKD-others: ADTKD individuals without UMOD variants, n = 5; ADTKD-suspected: individuals with suspected ADTKD, n = 11; healthy controls: n = 9. Data were expressed as mean ± standard deviation. In the cohort, ADTKD patients with a UMOD mutation expressed a lower plasma uromodulin concentration (7.05 ± 13.08 ng/ml) compared to patients without a UMOD mutation (70.3 ± 68.6 ng/ml). Both ADTKD-UMOD and the ADTKD-suspected (34.3 ± 28.0 ng/ml) group had a lower plasma concentration compared to healthy controls (153.5 ± 63.7 ng/ml).

We compared the clinical features of the probands with genetically confirmed ADTKD (N = 13) and clinically confirmed ADTKD-NOS (N = 5). Genetically confirmed ADTKD appeared to have a higher percentage of hypertension, but the other clinical features were similar between the two groups (Table 4). We also collected plasma from 6 ADTKD-UMOD, 5 ADTKD individuals without UMOD variants (ADTKD-others), 11 individuals with suspected ADTKD (ADTKD-suspected) and 9 healthy controls. The plasma uromodulin concentration in the ADTKD-UMOD group (7.05 ± 13.08 ng/ml) was statistically significant (p = 0.017) lower than the ADTKD-others (70.3 ± 68.6 ng/ml). Both of the ADTKD-UMOD and ADTKD-suspected (34.3 ± 28.0 ng/ml) groups had lower plasma concentrations compared to healthy controls (153.5 ± 63.7 ng/ml) (Fig. 3).

**Table 4:** Clinical features of the patients with ADTKD and ADTKD-NOS.

Character	ADTKD (genetically confirmed)	ADTKD-NOS (clinically confirmed)	P value
Number	13	5	
Male/Female	6/7	2/3	1.000
Age of diagnosis	31.5 ± 10.8	26 ± 7.5	0.318
Creatinine (μmol/L)	213.1 ± 91.6	177.9 ± 40	0.426
Uric acid (μmol/L)	531.6 ± 153.6	493.7 ± 156	0.645
Blood Urea Nitrogen (mmol/L)	8.8 ± 6.1	6.4 ± 3.8	0.425
24-h urinary protein (g)	0.19 ± 0.21	0.47 ± 0.56	0.328
With/without Proteinuria (No.)	2/11	1/5	0.099
Gout	4	1	1.000
Hypertension	6	0	0.114
Renal cyst	38.5%	40%	1.000
Family history	76.9%	100%	0.522
**Renal biopsy**	4	5	
Tubulointerstitial-nephropathy	4	3	
Medullary cystic kidney disease	0	2	
Cystic dilation of tubules/acute tubular injury	0	0	
Number of patients with genetic variation	13	0	

Statistic analyzed by independent-sample t test or Fisher exact test.

*Y* yes, *N* no, *ADTKD* autosomal dominant tubulointerstitial kidney disease, *NOS* not otherwise specified.

## Discussion

In the present study, we screened for variants in five disease genes known to cause ADTKD including *UMOD, REN, HNF1B, SEC61A1,* and *MUC1* in a Chinese cohort with suspected ADTKD. We identified 13 significant variants in this cohort. Eleven, one and one variants located in the *UMOD* (11/21 = 52%), *REN* and *HNF1B* genes*,* respectively. Previous studies of ADTKD in Chinese cohort were mainly case reports. Twelve variants in the *UMOD* gene have been reported in patients with ADTKD-UMOD^17^ and more than half of them were reported by our group^18–20^. Although the disease prevalence varied across different studies in different ethnic groups, a study with 136 probands from France showed that 17.6% and 3.7% variants were detected in the *UMOD* and *HNF1B* genes, respectively^15^. Another study from 56 Spanish families reported 16% and 28% variants in *UMOD* and *MUC1* genes, respectively^16^. The difference between groups may be due to the clinical evaluation of the suspected-ADTKD patients and that not all suspected patients accepted genetic testing. *MUC1* is another important disease-causing gene for ADTKD. Genetic testing for *MUC1* is challenging due to its complex VNTR region. Researchers have reported several methods to detect *MUC1* mutations, including snapshot, mass spectrometry and long-range PCR with NGS^3,7,8,21,22^. Early studies reported only one mutation of a single cytosine insertion in the *MUC1* VNTR region. Moreover, a recent study showed other novel frameshift mutations in *MUC1*^8^. In this study, we used long-range high-fidelity PCR and NGS to sequence the entire *MUC1* gene. Studies from Europe and America showed a varied prevalence of ADTKD-MUC1. ADTKD-MUC1 was reported to be rare in Chinese^23,24^. The clinical manifestations of hereditary cystic kidney disease were hard to distinguish, therefore, suspected ADTKD patient without ADTKD gene mutations may be due to other hereditary kidney diseases. Even though we performed WES in ADTKD-suspected patients with a family history in this Chinese cohort, we did not detect pathogenic variants in genes responsible for hereditary kidney disease. However, further investigation is warranted due to the limited number of patients enrolled.

Previous studies did not reveal specific differences between ADTKD-UMOD and ADTKD-MUC1 or ADTKD groups^16^. In this study, we also found that the clinical characteristics were similar between genetically confirmed ADTKD and clinically confirmed ADTKD-NOS patients. Family history of CKD or hyperuricemia suggested a higher detection rate for variants. In the sporadic individuals, the average age was much younger than those with a family history, which may be due to the inclusion criteria. However, in “sporadic” probands, we also indeed identified *UMOD* mutations, suggesting that genetic testing should be considered in young individuals with tubulointerstitial nephropathy.

Plasma uromodulin level is highly correlated with eGFR and lower uromodulin concentration associated with lower eGFR^25,26^. All individuals with ADTKD had lower levels of plasma uromodulin compared with healthy controls, which is consistent with the decreased eGFR in the ADTKD groups. It is noteworthy that the plasma uromodulin levels in individuals with *UMOD* variants showed a decreasing trend when compared to patients without *UMOD* variants in our study (Fig. 3). Individuals with *UMOD* variants often had a common feature of decreased secretion of uromodulin into the urine, due to the retention and accumulation of mutant protein in the endoplasmic reticulum^27,28^. The lower plasma uromodulin concentration in ADTKD-UMOD individuals may be caused by the accumulation of mutant uromodulin in the endoplasmic reticulum, decreased protein production or rapid proteolysis for degradation. Thus, the plasma or urinary uromodulin level may be considered as a marker to distinguish ADTKD-UMOD from other subtypes of ADTKD. Survival analysis indicated a better prognosis in ADTKD which may provide another clue to differentiate the ADTKD groups. However, further investigation with the inclusion of more clinical samples to reproduce these results is warranted to further demonstrate the clinical utility of this uromodulin biomarker found in plasma.

Up-to-date more than 100 variants in the *UMOD* gene have been identified, and 50% of them were cysteine replacement^29^. Researchers have raised the question regarding the relationship between prognosis and mutation type. A study showed that the individuals with a cysteine substitution had worse renal survival, however, it was a slight trend without significance (p = 0.41)^15^. In our cohort, 45.5% of variants lead to a cysteine replacement, similar to the previous report. Besides, individuals with cysteine substitution showed a better prognosis in our follow-up study. We also analyzed the relationship of genotype and outcome, based on the protein location of the variants. In previous reports, around 60% of *UMOD* variants were located in the D8C region, 30% in the EGF-like domains, and less than 10% in the ZP domain^17,29–34^. In this study, there was no obvious clustering among the three domains, even though more than half of the variants were located in the EGF-like domains. The results suggested that for this patient cohort the outcome of the ADTKD-UMOD group was associated with the type of amino acid substitution rather than the location of the variants. A larger cohort study is essential to replicate this genotype–phenotype correlation in ADTKD-UMOD.

Uromodulin is a glycoprotein with approximate 30% N-glycans and is suspected to be a factor that influences the prognosis of ADTKD. The N-glycans play an important role in uromodulin polymerization and defense against urinary tract diseases^35^. Abnormal uromodulin glycosylation was identified in renal transplant recipients and is associated with renal tubular injury as well as renal allograft rejection^36^. Some of the variants in the *UMOD* gene can influence the glycosylation process, or even change the binding sites of glycans directly. In this study, we only observed one residue that can change the potential glycosylation site (p. Asn38Ile, F3), and the person with this variation reached ESRD within 2 years.

In summary, we found gene variants in 61.9% of individuals in a Chinese ADTKD cohort and most of the mutations located in the *UMOD* gene. Family history was an important indication for further genetic diagnosis. Renal survival of individuals with ADTKD-UMOD was unrelated to the position of the variants. Direct sequencing of *UMOD* was the preferred method for ADTKD diagnosis. The plasma or urinary uromodulin level may be considered as a marker to distinguish ADTKD-UMOD from other subtypes of ADTKD. Even though WES in ADTKD-NOS patients with family history, we did not find other pathogenic variants in genes associated with hereditary kidney disease. However, further investigation is warranted due to the limited number of patients enrolled in this study.

## Materials and methods

### Study subjects and laboratory analyses

Forty-eight probands and other family members (N = 27) were enrolled in this study from 2012 to 2018. All participants accepted an informed consent form for genetic testing. Among them, 18 probands were diagnosed as ADTKD patients, based on combining genetic screening, family history, clinical features, and renal histological changes according to the KDIGO guideline (Table 1)^1^. The inclusion criteria are; (1) patients who have positive family history of chronic kidney disease, or early-onset hyperuricemia/gout compatible with ADTKD clinical characters; (2) patients without positive family history of CKD, must fulfill the clinical characteristics, or demonstration of compatible histology on kidney biopsy (interstitial fibrosis, tubular atrophy or dilatation, thickening and lamellation of tubular basement membranes, negative immunofluorescence for complement and immunoglobulins) or extrarenal manifestations compatible with HNF1B mutations. The clinical characters of ADTKD patients are autosomal dominant inheritance, progressive loss of kidney function, bland urinary sediment, absent-to-mil albuminuria or proteinuria, no severe hypertension during early stage, no drug exposure potentially causing tubulointerstitial nephritis, normal or small-sized kidneys on ultrasound, nocturia or enuresis in children. Patients were excluded if they have other causes of tubulointerstitial nephropathy, such as drug-induced or other knew genetic kidney disease. Estimated glomerular filtration rate (eGFR) was calculated using the formula of CKD-EPI. The definition of CKD was evaluated based on the KDIGO guideline (2012). The definition of ESRD was patient received renal replacement therapy or renal transplant. Moreover, the clinical data and family history of the patients were obtained from their medical records.

The first diagnosed ADTKD-UMOD patient was in 2012. Part of the protocol included follow-up. Individuals were interviewed by telephone or at an outpatient clinic. The follow-up study was focused on two aspects, treatment and progression of the disease. The primary endpoint was death or ESRD.

The study was approved by the local ethics committee of Peking University First Hospital (protocol No. 2016 [1133]). The study was performed in accordance with the Helsinki declaration and its later amendments or comparable ethical standards. All participants in this study signed written informed consent forms.

### Measurement of plasma uromodulin concentration

The plasma uromodulin concentration was measured with an enzyme-linked immunosorbent assay (ELISA) kit according to the manufacturer’s instructions (Euroimmun Medizinische Labordiagnostika AG, Germany).

### Screening for genetic variants

#### Sanger sequencing of the UMOD, REN, HNF1B and SEC61A1 genes

Genomic DNA was extracted from peripheral leukocytes derived from whole blood samples^37^. All of the coding and splicing regions of *UMOD* (NM_001008389), *REN* (NM_000537) and *HNF1B* (NM_000458) were amplified by PCR (Supplementary table S1) and Sanger sequenced with the ABI 3730XL Genetic Analyzer (Applied Biosystems). The reference sequences were obtained from Ensembl (Transcript number for *UMOD, REN, HNF1B* are ENST00000302509, ENST00000272190 and ENST00000225893). *SEC61A1* was a recently reported candidate gene for ADTKD^6^. We detected *SEC61A1* variants using the touch-down PCR program described by Bolar et al.^6^. Confirmatory testing was performed by bidirectional sequencing.

To validate variant pathogenicity, we sequenced DNA samples from 92 healthy controls and performed mutation and population database searches, namely, 1000 genomes (http://browser.1000genomes.org/), ExAC Browser (http://exac.broadinstitute.org/), human gene mutation database (http://www.hgmd.org/) and ClinVar (http://www.ncbi.nlm.nih.gov/clinvar). We utilized two software for pathogenicity predictions of novel variants, including Polyphen2 (http://genetics.bwh.harvard.edu/pph2/) and SIFT (http://sift.jcvi.org). Pathogenicity of the identified variations was evaluated using the ACMG criteria^38^.

#### Copy number variation of HNF1B detected by MLPA assay

Since gross deletions of the *HNF1B* gene can cause ADTKD^4,39^, we detected these types of copy number variants (CNVs) using multiple ligation-dependent probe amplification (MLPA) assays (SALSA MLPA P241-B1 MODY, MRC-Holland, Amsterdam, Netherland)^40^. With this method, all exons of *HNF1B* were amplified by PCR and assessed for CNVs inpatients and 10 healthy controls. Following MLPA, we performed capillary electrophoresis with ABI 3730XLGenetic Analyzer (Applied Biosystems) for fragment size determination and CNV detection using the Coffalyser.NET (MRC-Holland, Amsterdam, Netherland) software.

#### Long-range high fidelity PCR and next-generation sequencing for MUC1

Variants in *MUC1* were a challenge for NGS detection because of the VNTR region. In this study, we used long-range high fidelity PCR combined with NGS to overcome this challenge. A similar strategy was reported by Martina Zivná et al. previously^8^. Through PCR amplification with PrimeSTAR GXL DNA Polymerase (TAKARA BIO INC), The 4.7 kb product which covered the entire *MUC1* gene was acquired using previously designed primers. The PCR products were purified with Agentcourt AMPure X, followed by fragmentation to a library size of 350 bp. The library was constructed using the fragmented PCR products and the KAPA HyperPlus Library Preparation Kit. Moreover, quantification of the library was completed with the Library Quantification kit (KAPA, KK4824) and sequenced using the HiSeq (Illumina). Sequence reads were aligned to the reference sequence NM_001204286 and analyzed using NextGENE software. The *MUC1* mutation positive control was donated by professor Hong Cheng^24^.

#### Whole-exome sequencing

Whole exome sequencing was used in individuals with family history of ADTKD, but without identified variants in the 5 candidate genes. The positive family history was defined as family members with CKD, or hyperuricemia or both. One μg of DNA from probands was used to construct the exome library. Products were purified using AMPure XP system (Beckman Coulter, USA). DNA library enriched were sequenced on Illumina using paired-end 150 bp reads. The filtered reads were aligned to the NCBI human reference genome (GRCh37/hg19) by Burrows-Wheeler Aligner (BWA) software.

### Statistical analyses

Statistical analyses were performed with IBM SPSS Statistics package version 20. Quantitative parameters were presented as mean ± standard deviation. Qualitative parameters were presented as a number and percentage. Independent sample t-test was used to compare parametrical data (age, creatinine, uric acid, blood urea nitrogen, 24-h urinary protein) and chi-squared test or Fisher exact test was used appropriately to analyze the categorical variables. Kaplan–Meier analysis was used to analyze the prognosis. The results were considered statistically significant at p < 0.05.

## Supplementary Information

Supplementary Information

## Data Availability

The datasets generated and analyzed during the current study are available from the corresponding author on reasonable request.
