# Supplementary material for: Autosomal dominant tubulointerstitial kidney disease genotype and phenotype correlation in a Chinese cohort
Source: Sci Rep. 2021 Feb 11;11:3615. doi: 10.1038/s41598-020-79331-w (PMC7878898; doi:10.1038/s41598-020-79331-w)
Supplement: Supplementary file 1 — Supplementary Information [file 41598_2020_79331_MOESM1_ESM.pdf]

Autosomal dominant tubulointerstitial kidney disease genotype and phenotype correlation in a Chinese cohort

Kunjing Gong[1,2,3,4], Min Xia[1,2,3,4], Yaqin Wang[1,2,3,4], Na Wang [1,2,3,4], Ying Liu[1,2,3,4], Victor Wei Zhang[5,6], Hong Cheng[7], Yuqing Chen[1,2,3,4*]

1. Renal Division, Department of Medicine, Peking University First Hospital; Beijing 100034, China
2. Institute of Nephrology, Peking University;
3. Key Laboratory of Renal Disease, Ministry of Health of China;
4. Key Laboratory of Chronic Kidney Disease Prevention and Treatment, Ministry of Education;
5. AmCare Genomics Laboratory, Guangzhou, China
6. Baylor College of medicine Dept of Human and molecular Genetics, Houston, USA
7. Division of Nephrology, Beijing AnZhen Hospital, Capital Medical University, Beijing, 100029, China

*Correspondence to: Yuqing Chen, M.D. E-mail: cyq@bjmu.edu.cn, Renal Division, Department of Medicine, Peking University First Hospital; Institute of Nephrology, Peking University; Key Laboratory of Renal Disease, Ministry of Health of China; Key Laboratory of Chronic Kidney Disease Prevention and Treatment, Ministry of Education; Beijing 100034, China Telephone: (+8610)-83575525. Fax: (+8610)-66551055.

Supplementary figure S1. Flowchart of genotyping.

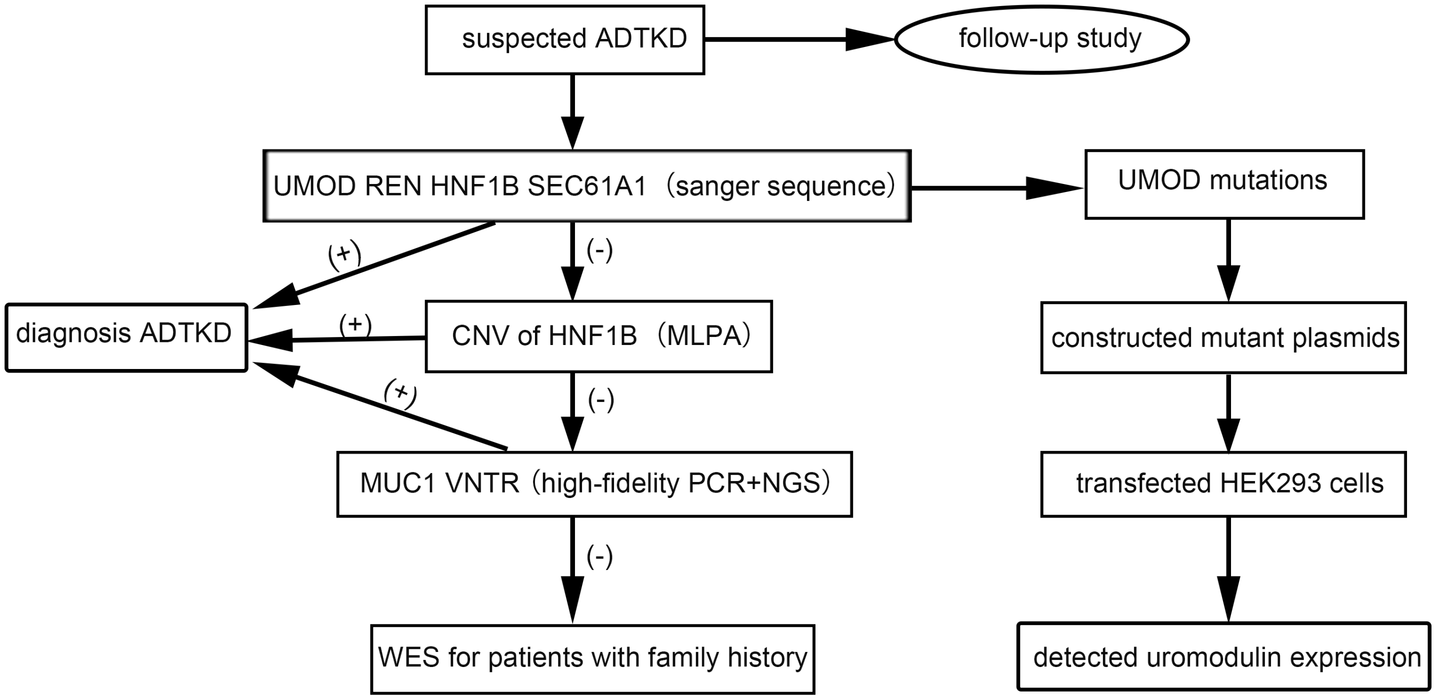

Supplementary figure S2. Schematic representation of uromodulin variants identified in the cohort

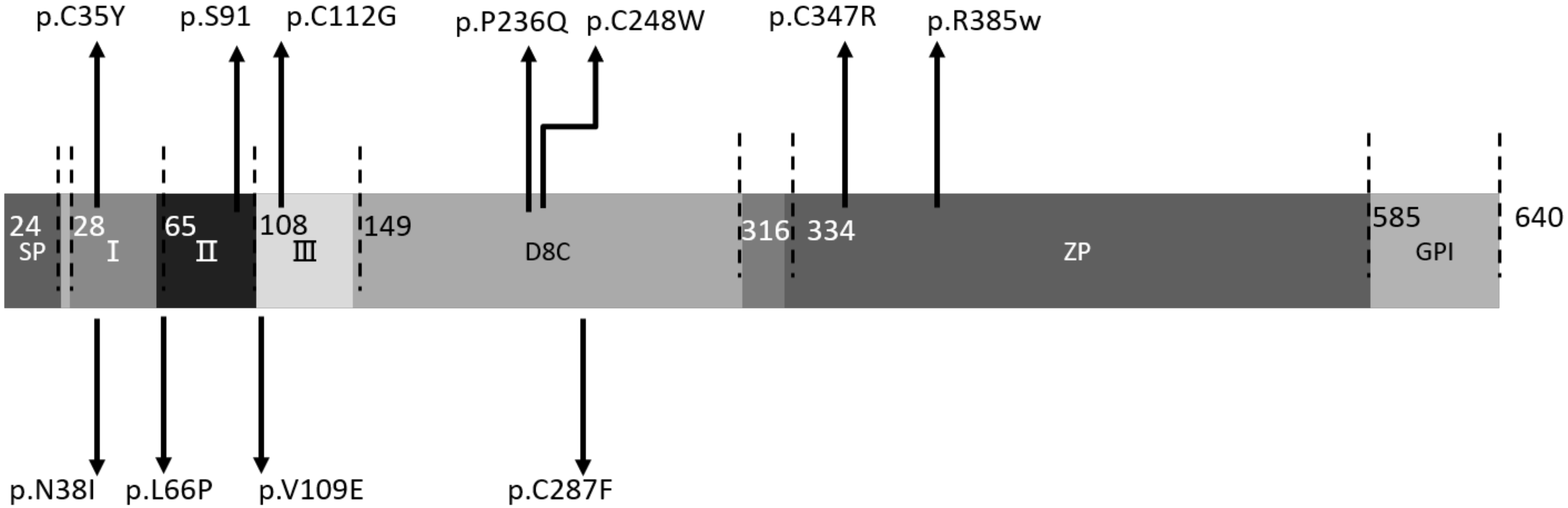

Positions of the 11 UMOD variants identified in the cohort are shown. The functional domains of uromodulin are from N-terminal to C- terminal, including N-terminal signal peptide, three epidermal growth factor (EGF)-like modules, a conserved cysteine residue (D8C), the zona pellucida (ZP) and a phosphatidylinositol (GPI) anchor. Half of the variations located in EGF-like regions. Variations in D8C and ZP were also found.

I: epidermal growth factor (EGF)-like I, II: epidermal growth factor (EGF)-like II, III: epidermal growth factor (EGF)-like III.

Supplementary figure S3. **HNF1B copy number variation detected by MLPA**

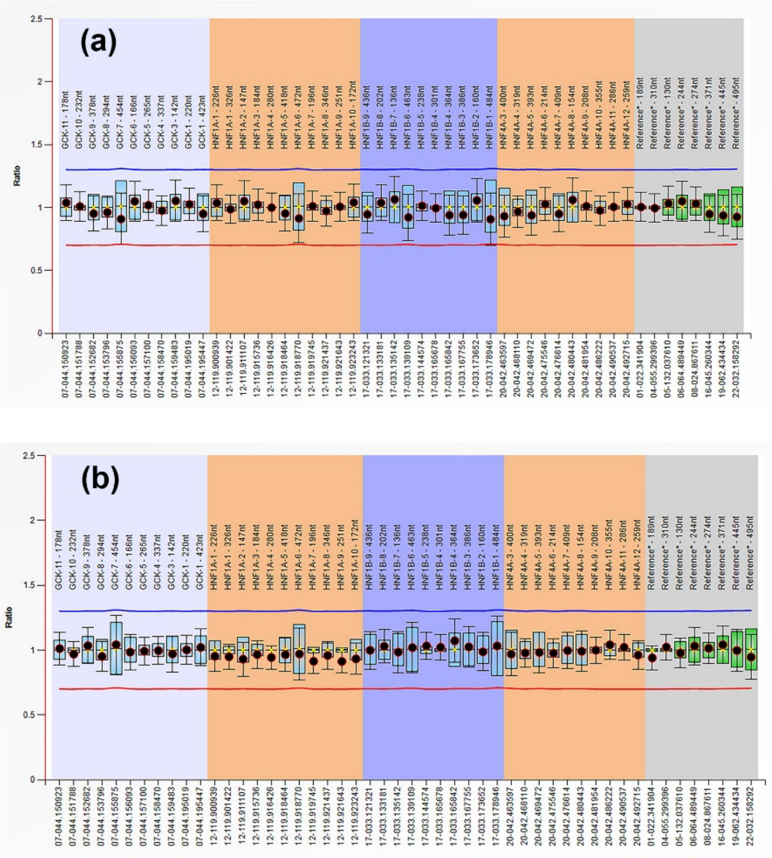

a: a healthy control, b: a patient. Data processing by CoffalyserNET(MRC-HOLLAND). Ratio of control and patient was closely to 1 which means no deletion or duplication of HNF1B exons.

Supplementary figure S4. Long-range high-fidelity PCR and next-generation sequencing for MUC1

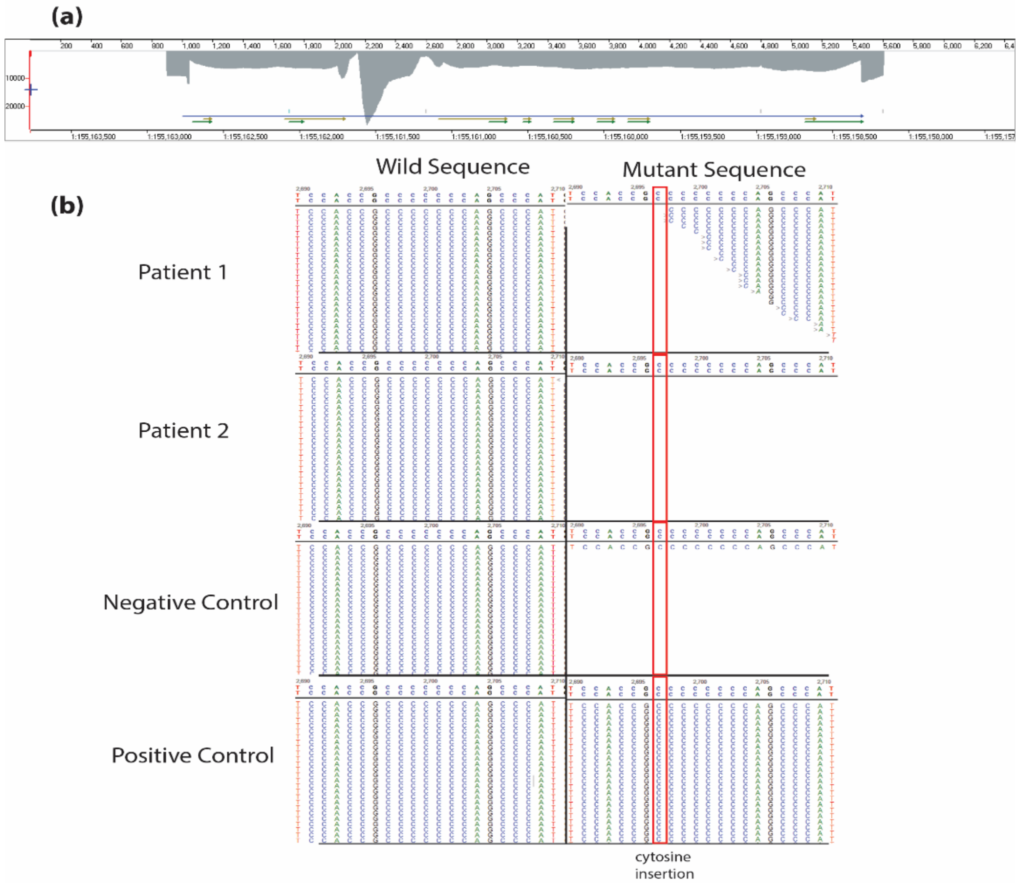

a: coverage of next-generation sequencing. b: data aligned with database. Two databases were designed with the wild-type gene sequence and the mutant gene sequence. All samples were aligned separately with the two databases using strict parameters. The sequence coverage of wild-type gene was above 1000×, and the mutant databases was above 100×, indicating positive samples. The red frame showed the position of cytosine insertion.

Supplementary table S1 Primer sequence of three genes

UMOD		REN		HNF1B	
EXON2	F:AAGGTGCGAAACAGTGACCT	EXON1	F:AAACCTGGGTACCCTTCACC	EXON1	Fa:TTCTTTTTCCGTCCTTGGAA
	R:CCCCAGTGTCCAAGGTCTTA		R:GCAAGGGGCATGTCCTAATA		Ra:CTGCGCCTACCTGAGCAT
EXON3	F:AAAACAAGGGCTGCAGTGTT	EXON2	F:CTCAGCGACAGAGGTGATTTC		Fb:GCCGGTCTTCCATACTCTCA
	R： CCCCCACACATTCACACATA		R:GATGCCTTCCTGTCTTCTGTG		Rb:GACTTCTCTGGTGGGAAACG
EXON4	F: CTGAAGCTGGGCTTTTCTGT	EXON3	F:GCAGGGTTAGGTTTGGAACTC	EXON2	F: GCCTCATGTCTACCCCAAAG
	R： GCCATCTGCCATTATTCGAT		R:CCTTGGTGGCTGTATCAGAAA		R: AGAGGGCAAAGGTCACTTC
EXON5	F: GAGTGTCACCTGGCGTACTG	EXON4	F:TGATGAAGCACATATTCCAAGC	EXON3	F: TCGTCCGTTGTCTGTCTGTC
	： GACTCAGGGAGCCTCAGAGA		R:GAGTCCCAGAGTCAACCTCAAC		R:AGGGTTCCTGGGTCTGTGTA
EXON6	F: AACCCACATTTAGGGGAACC	EXON5	F:ATTTCTGTACCCTGGAGCATGT	EXON4	F: CCCTTCATACTCCCAACCAA
	： TGATGAAACTGAGGCACAGC		R:AGCCACCAGTTGAATGCTTAAT		R: GCGTTTGCTCCTCTGAAAAC
EXON7	F:CCAGACATGAGACCAGCAGA	EXON6	F:AAGAGATGAGGTTCCAGCAGAC	EXON5	F: AGAGGTGCCGAGTCATTGTT
	R： GGGTTTGGGGTTAGAACCAT		R:GAGACAGAGAACTTCGGCATCT		R: TCTGGACAGCCCTCATTTTC
EXON8	F: CAGTTGGTGGGTTCCACTCT	EXON7	F:GACTACCCTGGAACAACCTCTG	EXON6	F: TGCCAAGGAATCGCTAAGTC
	R： TCCTCCATCCAAGTCCAAAG		R:TGTTGAGGCAGTGAGTAGAGGA		R: GTCGTGGGTGAGTTTGAAGG
EXON9	F:CCCAAAGTCACACAGCTGAA	EXON8	F:CCCACACTCAGGAAGGACAT	EXON7	F: TCCCATGGAATCTCCTGTGT
	R： CCTGCAGCTTAGGTTTCCTG		R:TGTGAGGTGAACAAGCGAAG		R: ACCCAGAGAGGGAAAGTGGT
EXON10	F: AGTGGCTTGCCTGAGATCAT	EXON9	F:CTTCGCTTGTTCACCTCACA	EXON8	F: GGAGATGGGAGCTATGGTGT
	R： CCACTTGCTCCCAGTTCTTC		R:AGGCATAGTGATGCCCAAAC		R: AACAACAGGGAGCCTCAGAA
EXON11	F: GAGTTGTGTGGGATGAGCTG	EXON10	F:TCATGTGCTGGGTATGGAGA	EXON9	Fa: GGTTGAGTTGGGCATCATCT
	： ACAGGTCCCACTGCAGAAAG		R:CTTCCCACCTGAGGTTGTGT		Ra:AGAGGACAAGGGGCTTCACT
EXON12	F: AGCCTAGACGAGGCAGCTTA				Fb: CGAACAAACTGATGCGAAAA
	R： CCGTAGGATCCTTAGCACCA				Rb:GAACCATGGCAGGGAAAGTA
EXON4	R':CTGTTGGCGGAGCAGTTGT				

All above exons were amplified using the standard PCR method. Cycling condition for UMOD amplification: 94℃ for 5 minutes followed by 35 cycles of 94℃for 30 seconds, 63℃ for 30 seconds, 72°c for 45 seconds and hold at 72°c for 7 minutes. Cycling condition for REN and HNF1B amplification：94℃ for 5 minutes followed by 35 cycles of 94℃for 30 seconds, 61℃ for 30 seconds, 72°c for 50 seconds and hold at 72°c for 7 minutes

Supplementary table S2 Variants classified by ACMG

No.	Gene	Nucleotide Change	Effect on peptide sequence	SIFT	Polyphen (damaging)	ExAc	1000G	Reference	ACMG	ACMG evidence
F1	*UMOD*	c.104G>A	p.Cys35Tyr	deleterious	probably	0	0	20	pathogenic	PS3,PS4,PM2,PM6,PP2,PP3,PP4
F2	*UMOD*	c.1039T>C	p.Cys347Arg	deleterious	probably	0	0	20	Pathogenic	PS1,PS3,PS4,PM2,PP3,PP4
F3	*UMOD*	c.113A>T	p.Asn38Ile	deleterious	probably	0	0	20	Pathogenic	PS3,PS4,PM2,PP3,PP4
F4	*UMOD*	c.197T/C	p.Leu66Pro	neutral	possibly	0	0	20	Pathogenic	PS3,PS4,PM2,PP3,PP4
F5	*UMOD*	c.272delC	p.Ser91	deleterious	NA	0	0	20	Pathogenic	PSV1,PS3,PS4,PM2,PP3,PP4
F6	*UMOD*	c.326T/A	p.Val109Glu	neutral	probably	1	0	18	Pathogenic	PS3,PS4,PP3,PP4
F7	*UMOD*	c.334T>G	p.Cys112Gly	deleterious	probably	0	0	This study, 41	Pathogenic	PS1,PS4,PM2,PP3,PP4
F8	*UMOD*	c.707 C/A	p.Pro236Gln	deleterious	probably	0	0	18	Pathogenic	PS1,PS3,PS4,PM2,PP1,PP3,PP4
F9	*UMOD*	c.744C/G	p.Cys248Trp	deleterious	probably	0	0	18, 42	pathogenic	PS1,PS3,PS4,PM2,PP1,PP3,PP4
F10	*UMOD*	c.860G.>T	p.Cys287Phe	deleterious	probably	0	0	20	Pathogenic	PS3，PS4,PM2,PP1,PP3,PP4
F11	*UMOD*	c.1153C>T	p.Arg385Trp	deleterious	probably	20	2	20	likely pathogenic	PS3,PP4
F12	*REN*	c.95A>C	p.Lys32Thr	neutral	benign	4	1	This study	uncertain significance	PP4,BP4
F13	*HNF1B*	c.884G>A	p.Arg295His	deleterious	probably	0	0	This study, 12	likely pathogenic	PS1,PM2,PP3,PP4

Supplementary table S3 WES of the individuals excluded the candidate genes of inherited kidney disease

**Inherited kidney disease**	**Gene**
nephrotic syndrome type 1-8	*NPHS1; NPHS2;PLEC1;WT1;LAMB2;PTPRO;DGKE;ARHGDIA*
FSGS1-6	*ACTN4; TRPC6;CD2AP;APOL1;INF2;MYO1E*
juvenile nephronophthisis	*NPHP1-18*
polystic kidney disease	*PKHD1; PKD1; PKD2*
Nephronophthisis	*INVS; RPGRIP1L; GLIS2; NEK8; TMEM67; TTC21B; WDR19; ZNF423*
Vesicoureteral reflux	*ROBO2; SOX17; TNXB*
Renal tubular dysgenesis	*AGT; AGTR1; ACE*
Joubert's syndrome	*INPP5E; TMEM216; AHI1; CEP290; TMEM67; RPGRIP1L; CC2D2A; OFD1; TTC21B; TMEM237; CEP41; TMEM138; TCTN3; ZNF423; TMEM231; CSPP1; PDE6D*
Meckel-Gruber syndrome	*MKS1; TMEM216; TMEM67; CEP290; RPGRIP1L; CC2D2A; TCTN2; B9D1; B9D2; TMEM231*
Bardet-Biedl syndrome	*BBS1-12; ARL6; MKKS; TTC8; PTHB1; TRIM32; MKS1; CEP290; LZTFL1*
Renal tubular diseases and metabolic diseases	*SLC families; CA2; PHEX; FGF23; ENPP1;DMP1; CTNS; CLCN5; OCRL; KCNJ1;CLCNKB; CLNCKA;BSND; CASR; TRPM6; FXYD2; CLDN16; EGF; CLDN19; CNNM2; KCNA1; SCNN1G; SCNN1B; SCNN1A; SCNN1G;SCNN1B; WNK1; WNK4; KLHL3;CUL3; KCNJ10; ATP6V0A4; SLC4A1;ATP6V1B1; AVPR2; AQP2*

WES was performed on 18 probands (4 clinically identified ADTKD and 14 suspected ADTKD). All of them had a positive family history of CKD, or hyperuricemia or both. None of the 18 individuals found the disease-causing genes of inherited kidney disease. This table showed part of the genes for differential diagnoses.

Supplementary table S4: Clinical features and outcome of the follow-up participants

No.	Family History	Gender	Diagnosis Age	Creatinine µmol/L	Uric acid µmol/L	Renal cyst	Interstitial Fibrosis	Treatment	Follow-up (year)	ESRD (yes/no)/age
F1	No	Male	20	163	749	No	NA	XO inhibitors	4	No/24
F2	No	Female	22	154	691	No	Yes	XO inhibitors	5	No/27
F3	Yes	Female	38	155	253	No	NA	No	2	Yes/40
F4	Yes	Male	44	203	380	Yes	Yes	BP-lowering drugs	5	No/49
F5	Yes	Male	50	317	414	Yes	NA	BP-lowering drugs	2	Yes/52
F6	No	Male	18	195	489	No	NA	XO inhibitors	2	Yes/20
F7	Yes	Female	41	250	540	No	NA	XO inhibitors	0	No/41
F8	Yes	Female	24	230	606	No	Yes	XO inhibitors +BP-lowering	2	Yes/26
F9	Yes	Female	21	136	433	Yes	NA	No	5	No/26
F10	Yes	Female	41	182	474	Yes	NA	BP-lowering drugs	4	No/45
F11	Yes	Male	22	469	781	No	Yes	XO inhibitors	3	Yes/25
F12	Yes	Male	34	158	617	Yes	NA	XO inhibitors +BP-lowering	1	No/35
F13	Yes	Male	34	158	484	No	NA	XO inhibitors+ antidiabetic drugs	4	No/38
F14	Yes	Male	33	150	633	Yes	Yes	XO inhibitors	2	No/35
F15	Yes	Male	35	152	580	No	Yes	XO inhibitors	1	No/36
F16	Yes	Female	32	117	451	No	NA	XO inhibitors	5	No

No.	Family History	Gender	Diagnosis Age	Creatinine µmol/L	Uric acid µmol/L	Renal cyst	Interstitial Fibrosis	Treatment	Follow-up (year)	ESRD (yes/no)/age
F18	Yes	Female	46	86	300	Yes	No	BP-lowering	5	No
F20	No	Female	28	128	379	Yes	Yes	No	4	No
F24	No	Male	22	289	531	No	Yes	XO inhibitors	4	No
F25	Yes	Male	13	77	521	No	Yes	XO inhibitors	4	No
F28	No	Female	19	140	259	Yes	Yes	No	4	No
F30	No	Male	17	230.3	600	No	Yes	XO inhibitors +BP-lowering	3	Yes/19
F33	No	Female	29	124	689	Yes	Yes	XO inhibitors +BP-lowering	3	No
F35	Yes	Female	47	149	208	No	Yes	BP-lowering	3	No
F37	No	Male	27	102	379	Yes	Yes	XO inhibitors +BP-lowering	3	No
F38	No	Male	13	123.9	653	Yes	Yes	XO inhibitors	2	No
F49	Yes	Female	26	166	514	No	Yes	XO inhibitors +BP-lowering	2	No
F41	Yes	Female	59	272	345	No	No	XO inhibitors	2	No
F43	Yes	Male	21	216	591	No	Yes	BP-lowering	2	No
F44	Yes	Male	55	111	386	Yes	Yes	BP-lowering	2	No
F45	No	Female	30	206	308	No	Yes	BP-lowering	2	No
F46	Yes	Male	24	143.2	479.8	No	NA	XO inhibitors	2	No
F47	Yes	Male	51	101	538	No	NA	XO inhibitors	1	No
F48	No	Female	20	132.8	576	No	No	XO inhibitors	1	No

F1-F15 ADTKD, F16-F48 ADTKD- suspected F: family; NA: not available; XO inhibitors: xanthine oxidase inhibitor, allopurinol or febuxostat; BP-lowering drugs: blood pressure lowering drugs; ESRD: end stage renal disease.

References

41 Dahan, K. et al. A cluster of mutations in the UMOD gene causes familial juvenile hyperuricemic nephropathy with abnormal expression of uromodulin. J Am Soc Nephrol 14, 2883-2893, doi:10.1097/01.asn.0000092147.83480.b5 (2003).

42 Wolf, M. T. et al. Mutations of the Uromodulin gene in MCKD type 2 patients cluster in exon 4, which encodes three EGF-like domains. Kidney Int 64, 1580-1587, doi:10.1046/j.1523-1755.2003.00269.x (2003).
